# Microplastic regulation should be more precise to incentivize both innovation and environmental safety

**DOI:** 10.1038/s41467-020-19069-1

**Published:** 2020-10-21

**Authors:** Denise M. Mitrano, Wendel Wohlleben

**Affiliations:** 1 Department of Environmental Systems Science, ETH Zurich, Universitätstrasse 16, 8092 Zürich, Switzerland; 2 Process Engineering Department, Eawag—Swiss Federal Institute of Aquatic Science and Technology, Überlandstrasse 133, 8600 Dübendorf, Switzerland; 3 Department of Material Physics and Analytics and Department of Experimental Toxicology and Ecology, BASF, Carl-Bosch-Strasse 38, Ludwigshafen, 67056 Germany

**Keywords:** Environmental sciences, Environmental impact, Materials science, Industry, Policy

## Abstract

The presence of plastic in the environment has sparked discussion amongst scientists, regulators and the general public as to how industrialization and consumerism is shaping our world. Here we discuss restrictions on the intentional use of primary microplastics: small solid polymer particles in applications ranging from agriculture to cosmetics. Microplastic hazards are uncertain, and actions are not similarly prioritized by all actors. In some instances, replacement is technically simple and easily justified, but in others substitutions may come with more uncertainty, performance questions and costs. Scientific impact assessment of primary microplastics compared to their alternatives relies on a number of factors, such as microplastic harm, existence of replacement materials and the quality, cost and hazards of alternative materials. Regulations need a precise focus and must be enforceable by these measurements. Policymakers must carefully evaluate under which contexts incentives to replace certain microplastics can stimulate innovation of new, more competitive and environmentally conscious materials.

## Abstract (web summary)

Plastic pollution is recognized as a global threat, but policy hurdles and a lack of effective plastic substitutes contribute to the problem. In this Perspective, the authors argue that an effective and sustainable path forward must rely on key restrictions and regulations optimized for impact and efficacy.

## Introduction

In recent years, stark images of plastic washing up on shorelines and accumulating in cities have underscored the magnitude of mismanaged plastic waste and its impact on the environment^1^. Solid nano- and microplastic fragments and fibers are part of the overall plastic pollution problem, as these small plastic pieces have been detected in every place that has been sampled so far^2–7^. Even though it is projected that the vast majority of these materials are derived from mismanaged waste in the environment^8^, legislation has been successfully passed in the US and UK, among other places, which bans microplastics used in rinse-off consumer products, such as microbeads in body scrubs^9^. These campaigns received widespread support, including from the public, scientists, and industry, as omission or substitution of these materials was relatively straightforward and inexpensive with alternate materials^10^. The trade-offs of using microplastics are not always easily identifiable or evaluated. In a number of contexts, the use of microplastics have been key in reducing environmental pollution (e.g. agricapsules for more targeted plant protection) and increasing human safety (e.g. replacing solvent-borne with water-borne coatings and adhesives). When attempting to balance the relative impacts of using microplastics versus opting for alternatives, there are several considerations one must confront including (1) what are the environmental and health impacts caused by microplastic use; (2) what alternatives currently exist or can be brought to market in the near-term which provide similar functionality as microplastics; (3) what is the cost of replacement materials (and to whom); and (4) can new substitutions be developed that perform better with less adverse outcomes. There are currently few quantitative numbers associated with these factors, and so decision-making on how and if to use microplastics can be both complicated and subjective.

Nevertheless, the framing of environmental risks by the media and scientists has affected the public perception of this emerging contaminant and the publics desire for quicker and more decisive action to be taken. Bans on cosmetics with microplastic beads in the US, Korea, and elsewhere have been one reaction to this call. The recent microplastic definitions proposed by ECHA (European Chemicals Agency) for a potential restriction of solid primary microplastics via the REACH regulation (EU law for Registration Evaluation, Authorization and Restriction of Chemicals) goes far beyond the original scope of the ban the microbead campaigns. By definition, polymers (and subsequently, plastics) are a heterogeneous group of compounds, yet, going against previous REACH protocols of regulating singular compounds, microplastics are considered collectively. While it is tempting to speak in broad terms about plastic pollution and to curb all usage in every context, including microplastic, there are many examples of when microplastics may help to reach other sustainability goals. Overarching chemical bans may be appropriate when there is clear and overwhelming evidence that targeted substances cause harm (e.g. DDT, CFC), but is this the case with plastic? In our view, technical bans on primary microplastics are not the route to significant reductions of microplastics in the environment. A major part of the issue can and must be prevented by proper (macro)plastic waste collection, ideally as part of the transition towards a circular economy. However, some sources of environmental microplastic, such a tire and road wear, will not be able to be reduced through this route. Therefore, we believe it would be prudent to take a more nuanced approach to assess when and where plastic restrictions are most warranted.

In this perspective, we assess the scope, effectiveness and utility of microplastic regulations with specific emphasis on the new definitions proposed by ECHA for restriction of primary microplastics under REACH. To this end, we aim to (1) provide a systematic orientation of the polymer universe, to appreciate which (micro)plastic characteristics are relevant, measurable, and enforceable, (2) cluster-specific uses of solid plastic to highlight how primary microplastic can add to issues of environmental pollution and human health, (3) delineate the social and corporate drivers which prompt informal norms and formal regulations, (4) evaluate the strategy and effectiveness of new regulations that attempt to limit microplastic release, and (5) suggest priority cases where regulations should be focused and precision increased to incentivize innovation of sustainable materials and promote environmental health and safety.

## The diversity of polymers through their life cycle complicates microplastic risk assessment

Contamination of the environment with plastic debris is one of the major environmental challenges that affects society today^11,12^. Since the mass production of plastics began in the 1940s, manufacturing techniques have been optimized, resulting in a plethora of lightweight, durable, persistent, and corrosion-resistant plastic varieties^13^. These attributes have led to the extensive use of plastics in an inexhaustible number of applications^14^. The plastic materials differ in their life-cycle paths through production, use, recycling, or disposal. Closing all plastic life cycles towards circularity (e.g. circular economy, recycling, chemcycling) can be one way to limit environmental, and subsequently health, burdens of these materials. However, plastic leakages along the life cycle are still occurring today in large quantities and thus we need to understand how both the plastic items, as well as any incorporated additives and chemicals, affect environmental and biological systems^15^. The many diffuse sources of plastic, including microplastic, are generally anticipated to negatively impact the water quality of oceans, lakes, rivers and soil, but efforts are still under way to understand how different microplastic varieties will behave in and affect these ecosystems. Evaluating the relative sources, mobility and effect(s) of microplastic in these environments can help determine their associated risk(s) in the environment. To increase clarity on commonly used terms for materials and material properties, as well as regulatory bodies and tests, we provide a simple glossary (see Box 1).

Box 1 Glossary of terms **ECHA** European Chemicals Agency (EU)

**REACH** Registration, Evaluation, Authorization and Restriction of Chemicals

**EPA** Environmental Protection Agency (USA)

**ECETOC** European Centre for Ecotoxicology and Toxicology of Chemicals

**OECD Test Guidelines** Developed by the organization for Economic Co-operation and Development (OECD), these guidelines are a collection of the most relevant internationally agreed test methods used by government, industry and independent laboratories to determine the safety of chemical and chemical preparation

**ISO test method** From the international standards organization (ISO), various methods exist as an international standard for testing laboratories created to ensure laboratories are producing precise and accurate test data

**Enforceable** breach of a regulation can be evidenced by robust measurements

**Polymer** IUPAC: "Substances composed of macromolecules, very large molecules with molecular weights ranging from a few thousand to as high as millions of grams/mole.” OECD: "A polymer means a substance consisting of molecules characterized by the sequence of one or more types of monomer units and comprising a simple weight majority of molecules containing at least three monomer units which are covalently bound to at least one other monomer unit or other reactant and consists of less than a simple weight majority of molecules of the same molecular weight. Such molecules must be distributed over a range of molecular weights wherein differences in the molecular weight are primarily attributable to differences in the number of monomer units. In the context of this definition a 'monomer unit' means the reacted form of a monomer in a polymer,"^24^

**Polymers of low concern (PLCs)** Polymers which have been deemed to have insignificant environmental and human health impacts as assessed by the US-EPA and most other non-EU jurisdictions

**Plastic** A synthetic material made from a wide range of organic polymers such as polyethylene, polyvinyl chloride, nylon, etc., that can be moulded into shape while soft, and then set into a rigid or slightly elastic form. (https://en.oxforddictionaries.com/definition/plastic)

**Plastic additive** A substance which is intentionally added to plastics to achieve a physical or chemical effect during processing of the plastic or in the final material or article; it is intended to be present in the final material or article.”^16^

**Bioavailability** the fraction of an ingested substance that reaches systemic circulation

**Biodegradable plastic** A certified performance characteristic of plastic (tested e.g. by ISO 17088:2012) which is not dependent on the origin of raw materials, but only on the chemical composition of the polymer. Biodegradability of a plastic is intended and tested for one specific environmental compartment

**Biobased plastic** plastics which are based on biological feedstocks, such as lignin or corn, to replace petroleum feedstock for polymer synthesis. Biobased plastic is not necessarily biodegradable

**Green Chemistry** the practice of designing chemical products and processes with reduce or eliminate the use or generation of hazardous substances

**REACH concept of nanoforms** Distinguish different “nanoforms” of the same substance by differences in particle size, particle morphology, particle surface treatment, crystallinity and specific surface area^31^

**Solubility** Maximum mass of a material that is found in a molecularly dissolved state in a given volume of water containing a particulate material under specific conditions

**Functional groups** an atom or association group of atoms in a chemical substance that is intended to or can be reasonably anticipated to undergo facile chemical reactions

**Bioavailability** The proportion of an ingested substance which enters the circulation of an organism and so is able to have an effect

**Risk assessment** the probability of an adverse effect on humans or the environment occurring as a result of a given exposure to chemical or mixture

**Trojan horse effect** Also known as carrier effect; the transport of pollutants while carried by particles

**Frustrated phagocytosis** The inability of macrophages to engulf objects longer than approximately 15 μm. This is particularly noted in the case of long, still fibrous substances such as asbestos and, likely, microplastic fibers

### A tour of the polymer universe

**Fig. 1:**
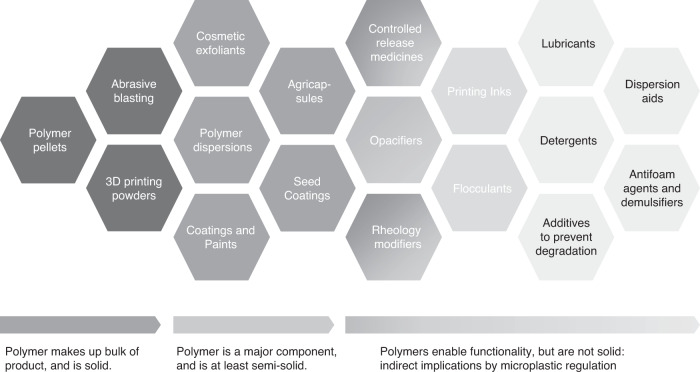
The universe of polymer species from primary microplastics to functional polymers, where solidity decreases, and solubility increases from polymer pellets to dispersion aids. The appropriate scope of microplastic regulation is a matter of open debate especially on the semi-solid polymer species, and approaches differ regionally (Table 1).

Polymers and plastic exist on a spectrum from solid particles (e.g. primary microplastics) to soluble functional polymers. It is upon these physical characteristics (solidity, solubility, mass percent of particles, etc.) on which regulations are currently based. Figure 1 sorts all species of primary microplastics by decreasing solidity to increasing solubility from black to gray, and continues into the universe of functional polymers, which are liquid or soluble and depicted in light gray. Some of the borderline cases of relatively soft and swellable particles are shaded gray to light gray. The term microplastic is generally used to describe the issue, the products, and their regulation, though one might argue that particulate plastic is a more precise framing of the specific issue and of the regulation of solid, insoluble plastic particles since the size range of the materials extends beyond the microlength scale. These materials may incur environmental releases, e.g. controlled release medicines or rheology modifiers in cosmetics. The light gray species are typically of lower molar mass and higher functionalization, resulting in absence of solidity, absence of particle shape, and increasing solubility. Functional polymers (light gray in Fig. 1) are generally not considered as microplastics. The physical properties of plastics and their use in final products and formulations partially influence how they may enter the environment. Taken as a whole, a one size fits all regulation will not be able to encompass the universe of polymer species because of the diversity of materials which exist.

### Plastics in the environment: what, where, why, how much

**Fig. 2:**
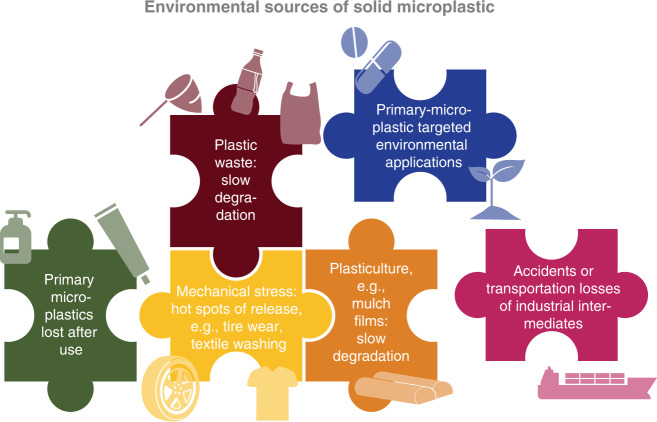
Environmental sources of pollution by microplastic. Some sources and pathways are interconnected (e.g. mechanical stress, plastic waste, plasticulture) and some sources are stand-alone (e.g. primary microplastics in products, targeted applications, or transportation losses), but collectively all sources are part of the puzzle of how microplastic enters the environment.

Plastics can be lost to the environment across their entire value chain, which creates different opportunities (and challenges) to prevent leakage into technical and natural systems^17^. In this context, it is useful to frame the separate but interconnected issues of plastic pollution, which are nestled into one another (Fig. 2). This list of microplastic sources entering the environment is oriented on a probable ranking of relevance in terms of mass of plastic lost through a given route^11,18,19^, which ultimately varies by region^20^. While evidence on the absolute number or mass of microplastics which originate from each source is very limited by lack of field measurements, based on our understanding of the production quantities, and mass flows^18^ we suggest the following:

1. Uncollected plastic waste, allowing slow degradation over time (e.g. mismanaged packaging, fishing nets).
2. Mechanical stress, creating hotspots of high release instances (e.g. tire wear, fibers from washing of textiles).
3. Plasticulture losses, allowing slow degradation over time (e.g. uncollected mulch films).
4. Primary microplastic for targeted environmental applications (e.g. seed coatings, fertilizer capsules).
5. Primary microplastic that is lost through consumption (e.g. cosmetic beads, drug delivery).
6. Accidents and transportation losses of industrial intermediates (e.g. plastic pellets, polymer dispersions).

Through tackling the mismanagement of plastic waste, we can reduce general macroplastic pollution and prevent microplastic pollution from secondary microplastic forming in-situ (sources 1 and 3)^20,21^. In contrast, the contribution of tire wear is hard to avoid (source 2), although it is suggested to be one of the dominant sources of microplastics in Europe^22^. For plasticulture (source 3), we anticipate that recollection will never be loss-less, such that we should redesign current products to biodegrade in the environmental compartment where they are used. However, we do not mean to imply that biodegradability of plastic is an excuse for littering, since biodegradation is designed for specific conditions, e.g., compost or soil; the same material may degrade slowly in freshwater or in the ocean. In this way, biodegradable plastic will not be a universal solution to reduce mismanaged plastic waste (source 1).

Primary microplastics are a smaller source of environmental contamination in comparison^22^, and can contribute to plastic pollution either by targeted applications in the environment (source 4) or losses through consumption of consumer goods (source 5). The chemical compositions for primary microplastics that contribute to sources 4 and 5 are highly diverse, and include acrylic copolymers, polyamides, polyurethanes, among others, and are thus chemically distinct from the composition of the most dominant secondary microplastics. Conventional plastic parts rely on polymer pellets on the order of 3 mm; more innovative technologies such as additive manufacturing involve micronized polymer particles on the order of 50 µm. Both are intermediate products that lose their particle nature during processing (injection molding or selective laser sintering, respectively). These primary particles are not released into the environment, except for source 6, accidents and transportation losses. The ranking in the impact assessment of the ECHA draft restriction is consistent with the ranking above^23^.

### Assessing impacts across the diversity of polymers

Primary microplastics currently make up a small fraction of total plastic pollution, but this alone is not enough to suggest that regulatory attention is inappropriate. Small quantities of a substance still have the potential to cause negative impacts. However, this strikes at the heart of the matter, in that in our opinion current (human or eco) toxicity data have not defined which polymer or plastic properties (size, shape, chemical composition, additives, etc.) are responsible for inducing harm which is specific to microplastics. There are some plastic-specific issues which are not accounted for in the current particle hazard assessment paradigms. The concept of hazard testing itself is fundamentally challenged by the composition of microplastics, with plastic having low water solubility, hence low bioavailability of the polymers, and by the particle size. For example, there are very few OECD Test Guidelines for either chemicals or particles which are applicable to particles of 500 to 50 µm in diameter, and none that reach the highest thresholds of the microplastic definition (5 mm). At the very least, the test organism has to be larger than the test item. This is not the case for a wide spectrum of microplastics compared to, e.g., *Daphnia magna* or algae in general which are considered as a key model of toxicity to aqueous species, or compared to bacteria or cell cultures used to test genotoxicity. If one applies the recent ECETOC (European Centre for Ecotoxicology and Toxicology of Chemicals) polymer risk assessment framework on potential ecological and human health hazards and risks posed by polymers, the large size of microplastic particles (far above the regulatory cut-offs at 1000 g/mol and at 10,000 g/mol molar mass that are critical to suppression of bioavailability), and the low reactivity of thermoplastic solid polymers (having no functional groups, and no redox centers) rules out mutagenicity, systemic effects, and local effects on skin and eyes, but leaves the need to assess pulmonary effects if inhaled^24^. The World Health Organization^25^ recently published a report suggesting that there were currently no overt health concerns associated with the exposure to microplastic particles through drinking water, but increasing concentrations of plastic in waterways and/or more study may reveal effects in the future^26^. Nanoplastics, which are more likely to cross barriers, are close to the molecular range of individual, water-swollen polymer chains. Therefore, we believe that the line between effects by microplastics and any other polymer effects may begin to blur for the size fraction below 100 nm. In reviewing the literature for more targeted or chronic effects, particularly for nanoplastics, Lehner et al.^27^ found that in vitro studies using human cell lines showed evidence that particles are taken up and induce oxidative stress or pro-inflammatory responses. It must be remembered that in the last 15 years, polystyrene spheres of approximately 100 nm in size have been used as “control” particles when testing substance-specific effects of inorganic nano-sized materials^28,29^. We believe that direct adverse effects to humans, even from nanoplastic, have not yet been identified as a consequence of ingestion from the food chain or from other environmental sources. In contrast, fibers may fall under the fiber paradigm of frustrated phagocytosis when inhaled, leading to fibrosis and lung cancer, if they are sufficiently rigid and long. Polymer fibers that are respirable starting at a few microns in diameter are worthy of  investigation for their potential for pulmonary toxicity^30^.

### Bridging assessment of microplastics with other particles and chemicals

We anticipate that the existing body of work on the hazards of particle exposure and corresponding regulatory guidelines for engineered nanomaterials may be partially applicable in relation to microplastic. For small particles (e.g. on the order of 1 µm), methods can be applied that were developed in the recent update of particle toxicology to assess engineered nanomaterials (Supplementary Table 1). In this regard, the level of detail of regulatory scrutiny has been dramatically increased by the REACH concept of nanoforms, where different “nanoforms” of the same substance are distinguished by differences in particle size, particle morphology, particle surface treatment, crystallinity, and specific surface area^31^. Screening methods of biological similarity concerning fate and hazard have become more important than ever. Through the development of OECD Test Guidelines or Guidances, testing protocols have become increasingly standardized. Some of the practical knowledge of testing particles and particular test systems will likely prove relevant when assessing nano- and microplastic impacts (see Supplementary Information and Supplementary Note). In the last years, significant work has begun in tackling the question of how microplastics interact with organisms and/or how they impact their fitness, but with little overarching consensus considering the variety of organisms, conditions, and materials tested^32^.

Several reviews have concluded that a nano-specific toxicity does not exist, in the sense that similar adverse effects were not demonstrated by all particles of similar size. Rather, it was shown that the composition of the particle was the most important parameter for toxicity^33,34^. Consequently, REACH dossiers cover all forms of a substance and, since January 2020, additional properties such as the dustiness (EN17199) and the dispersion stability in aquatic media (OECD Test Guideline 318) need to be described when registering nanoforms (Supplementary Table 1)^31^. When these concepts are extrapolated to small solid polymer particles, the different material properties of polymers compared to other nanoforms may no longer make REACH reporting requirements justifiable. For example, the surface reactivity and inflammation potential of plastics compared to inorganic metal-oxide materials is negligible^35^, and hydrophobic attachment reduces the dispersion stability of most microplastics.

Although the bioavailability of macromolecules above 10,000 g/mol is low, the leaching of low-molar-mass additives in plastic may induce relevant hazards. These additives may either be associated with the plastic from the production process (e.g. intentionally added compounds, such as UV stabilizers or non-intentionally added substances and byproducts^36^), or sorb to the particles once in the environment (e.g. persistent organic pollutants (POPs), via the putative Trojan horse effect). The leaching behavior of additives and subsequent negative impacts is potentially a property which could be tested across plastic types and conditions^37^, but the net contribution of the Trojan horse effect to hazard was measured to be minor^38,39^, considering (1) the similarity of the affinity of POPs to either polymer or lipids, enabling either net contamination and net decontamination activity and (2) the far higher contribution of natural particles, such as black carbon, to POP transport and ingestion^40^.

In review of the literature, we have found sparse knowledge for both dietary and airborne exposures, and little to nothing on the kinetics and biodistributions of particulate plastic. Considering the array of materials in question, environments to consider, biological and abiotic impacts to organisms of different trophic levels, as well as potential sources to humans (food, personal care products, city dust), we find it understandable that there has not yet been a convergence on which aspects of microplastics are most harmful.

## How are policy developments and industrial practices impacted by consumer voice and behavior?

Civic engagement on the topic of reducing plastic pollution in the environment, and curbing plastic use generally, is an extension of the responses to tackle global environmental problems that began in the late 1960s and early 1970s. The media is a vital link which connects science with the public and has the ability to heighten awareness of select topics. The newsworthiness of some topics may prompt increased attention on certain environmental issues, including how frequently and in which light the public views certain environmental risks^41,42^. How mass media and social media portray environmental issues ultimately plays a crucial role in framing scientific knowledge in the public eye, since the public does not always have access to, or cannot easily interpret, primary scientific sources. In this way, journalists and influencers play a role in defining the scope of a problem and guide non-experts to reach conclusions which are more easily digestible. However, we find that this often oversimplifies the complexities and uncertainties which scientific findings contain. Risk in the context of chemical assessment can be defined from the perspective of natural sciences as the probability of an adverse effect on humans or the environment occurring as a result of a given exposure to a chemical or mixture^43^. Therefore, a risk is the chance (high or low) that an exposure will actually cause harm. Microplastic risk assessment suffers from knowledge gaps on both hazard and exposure evaluations, which allows for the possibility of a few point data sources to dominate narratives. This absence of evidence does not allow one to conclude whether risk is either present or absent with any certainty. Public risk perceptions typically differ from experts’ assessment of risk^44^, particularly because the latter group tend to conceptualize risks in a formal way based on the seriousness of potential negative consequences. By omitting the important aspect of probability in evaluating adverse outcomes, both scientists’ and the publics’ responses to risks can become amplified^45^. Furthermore, concentration dependency of (microplastic) impacts is often given relatively little attention, both in academic papers and in the public media.

### From concern to action: coordinated efforts to drive real (or perceived) change

Independent of any one specific source or risk of plastic, the visual presence of litter in the environment and popular broadcasting (e.g. Blue Planet II by BBC) has prompted an evocative reaction for the public to demand action plans to reduce plastic consumption and waste. With the scale and prominence of plastic pollution, motivated actors (ecologically minded individuals, environmental NGOs) aim to disrupt established social behavior to promote change. Normative dynamics (norms) are studied in many areas of global governance, including environmental politics and environmental norms in areas such as climate change^46^. Just as there are multiple scales on which to address plastic pollution, so too are there multiple reactions the public has towards plastic in the environment which influence their demands for action to reduce plastic use. In addition to macroplastic reduction (e.g. plastic bag bans)^47^, grass-roots activism from environmentally conscious consumers, activists, and scientists led to successful lobbying for legislation to restrict the use of microbeads in rinse-off consumer products and other microplastic containing products in different regions (Table 1)^48^. While various bans differ in terminology and scope, in reviewing the impacts of the Microbead Free Waters Act in the United States, McDevitt et al.^49^ noted that the lack of appropriate standards for environmentally safe materials led to overarching bans which left stakeholders on all sides of the issue potentially dissatisfied. When looking at differences between the causes and reactions to macro- and microplastic pollution, we see that norms against plastic do not converge towards one shared problematizing of plastic. In contrast to other environmental regimes that arrange diverse instruments of governance around one shared goal, plastic governance lacks such a common narrative as an anchor for the diverse activities to reduce the environmental burden of plastic^50^.

**Table 1:** Examples of current regulations for polymers, additives and primary microplastics around the globe.

	EU	US	China	South Korea
Regulation of polymer, including functional polymers and solid plastics	Exempted from REACH due to low bioavailability	Polymers that fulfill the polymer of low concern criteria (PLC) are exempted from TSCA-submission of Pre-Manufacturing Notice, and can be commercialized	Apply new substance notification for import and manufacture if non-IECSC listed. The concept on Polymers of low concern is applied for simplified notification	Since 2019: K-REACH registration started, tiered approach based on tonnage when manufactured or imported
Regulation of additives	REACH, if >1 ton manufactured or imported	Additives are chemical substance which are subject to TSCA	Apply new substance notification for import and manufacture if non-IECSC listed	K-REACH registration, tiered approach based on tonnage when manufactured or imported
Regulation of primary solid microplastics	Proposed REACH restriction, potentially from 2022: banned if dispersed in environment labeling & reporting if used only industrially and/or losing particle nature in application biodegradable, or natural, or soluble polymers exempted	Use as scrubbing beads in cosmetics are banned on federal level (“ Microbead Free Waters Act”).Additional regulation(s) at state level is established or under way	General plan to prohibit “Microplastic” manufacturing after 31 Dec. 2020, and for sale after 31 Dec. 2022	Cosmetics Act prohibits scrubbing beads in cosmetics. Further microplastics issues are under discussion for polymer types linked to pollution via waste

*REACH* Registration, Evaluation, Authorization and Restriction of Chemicals, *TSCA* Toxic Substances Control Act, *IECSC* Inventory of Existing Chemical Substances in China, *K-REACH* Act on Registration and Evaluation of Chemicals in South Korea.

The consumer industry, which can leverage selling products via emotions, was quick to adapt to consumer preferences. Take for example the various washing bags and devices/objects aimed at removing microplastic fibers from the wash water over the course of laundering textiles. These marketed technologies (e.g. Cora Ball, GUPPYFRIEND, Lint LUV-R) claim to ultimately reduce microfiber emissions in the wash water effluent through various means, but their efficacy is uncertain^51^. We further generally question the logic (and sustainability) of producing more consumer goods to capture microplastics in individual households. Are the environmental costs in terms of material, energy and other resources to produce these products outweighed by the avoidance of microplastic fibers which are retained during washing? We feel that while this may provide consumers with a feel-good mentality that they are protecting waterways from microplastic fibers, the cost/benefit analysis of these products are debatable. However, consumer initiatives are essential to reduce unconscious emissions. More research should be done into effective and measurable reductions of (micro)plastic leakage to the environment through education, regulation, and behavioral change, possibly supported by new technology.

Corporate sustainability is an increasingly important goal which is being implemented at high levels across a range of industries. Through these initiatives, industry can also play a role in addressing global challenges such as e.g. reaching the United Nations sustainability goals. The growing interest in corporate sustainability can largely be attributed to the increased prevalence of global problems (e.g. climate change, plastic pollution) and a shift in public perception that firms should, at least in part, provide solutions to those problems^52^. Most large corporations engage in sustainability initiatives, report on environmental and social matters in annual reports, and publicize their efforts via social media. Yet to date the concept of corporate sustainability remains vague and tools to measure if/when an impact has been successful—as opposed to greenwashing—are non-existent^52^. Corporations can conform to plastics norms since doing so enables them to maintain legitimacy in the face of competing demands, expectations, and pressures, but environmental outcomes of “Green Alliances” between companies and NGOs are limited because rules are flexible, open, and not obligatory^53,54^. In the case of microplastics, without policy restricting certain materials and setting goals for a long-term sustainable development (e.g. by favoring replacement technologies, banning certain additives, or restricting polymer use), when all other factors are considered equal, we anticipate that industry is likely to favor short-term wins: no change or superficial changes to satisfy consumer emotions. In the context of microplastics, NGOs have begun to compile lists of products which do not contain microbeads, such as Fauna and Flora’s “Good Scrub Guide”, or to promote certification schemes which include product labeling, such as “Zero Plastics Inside” from the Beat the Microbead campaign by the Plastic Soup Foundation. While the stated goals of these well-intentioned programs are often to educate consumers rather than to explicitly change market dynamics, these schemes offer a platform for companies to be recognized as transparent, environmental leaders. Consumer industries wish to offer a feel-good premium to consumers and amplify their tendencies of purchasing goods and services which resonate with their perception of making positive, ecologically conscious choices. More examples of this nature exist in regard to macroplastic waste and initiatives as well. Adidas partnered with the Parley initiative in 2015 to offer shoes made from ocean-clean-up plastics. More than 15 million pairs sold in 2020 (ref. ^55^)—compared with 400 million Adidas shoe sales total—are testimony to clever marketing. While this may have also catalyzed the advancement of recycling technologies in some respects, creating shoes out of recycled plastic to reduce the pollution issue cannot be generalized: the costs of raw materials are a minor factor in the fashion industry, but are decisive for high-tonnage applications.

We think that the decisive element in truly changing industry and consumer behavior in relation to plastic usage and handling is cost: up until 2018, plastic biodegradability was a nice-to-have for consumer industries if functionality and price were not compromised. Since 2019, the consumer goods industry is willing to pay more to their chemical suppliers for biodegradable, recycled or bio-based plastics than for conventional plastics. Most recently, Nestlé committed 1.5 billion Swiss Francs to pay a premium until 2025 for new sources of packaging^56^. This compensates the costs of advanced plastic recycling technologies as required for food-grade plastics, which are currently higher. Another contribution can be expected from the “Alliance to End Plastic Waste”, funded with $1.5 billion to advance infrastructure in waste management, innovation in recycling, education of behavior and clean-up of plastic pollution hotspots. The Ellen MacArthur initiative is making progress in partnering with industry and governments on transitioning to a circular economy for plastic^57^. Different regions set different priorities, where the Europeans prioritize biodegradability, the US and Chinese consumers prioritize bio-based resources. Thus, consumer attitudes incentivize industry by creating secondary standards, even before legal standards force both consumers and industry to redirect the flow of materials.

These examples show the power of environmental norms in the dynamics and governance of plastic use and legislation, but the ability to shape these norms and policies are not shared equally geographically or across socio-economic classes around the globe^50^. Assessment of norms and their influence on driving regulations for plastic in Europe and the Americas can be illuminating to appreciate the factors that go into environmental governance. Nevertheless, in the case of attitudes towards plastic use, global norm diffusion patterns are often exemplified by liberal or Western ideals and global norms must be interpreted against very different local or national backgrounds and value systems. The market demand for sustainable plastics is likely to be driven primarily by higher income earners with more disposable income. In this regard, we interpret it that more wealthy, industrialized regions are often over represented in forming these global (plastic) initiatives with low- and middle-income economies, particularly those in the Global South, playing less of a role in the formation of global environmental norms. Environmental values can differ according to specific locations, and this may present competing, rather than complementary, steps to take when addressing the various pressing environmental (as well as societal and economic) concerns a region is pressed with. However, the heterogeneity of policy approaches to phase out lightweight plastic bags shows that income is not the only factor in regional differences^58^.

## How are (new) regulations stepping in to address these demands?

Plastic pollution is an undeniable ethical issue, because the accumulation of waste in soil and water is not sustainable for the planet, and it seems that public opinion holds quite some sway in pressuring political leaders to take action in drafting (micro)plastic regulations. Partially based on the public demands to address the ever-growing issue of plastic pollution, governments and organizations have been actively addressing these concerns through the development of new sustainability initiatives and regulations. The relatively swift creation of the European Union Strategy for Plastics in a Circular Economy is one example of this. On one hand, this helped to expose the many shortcomings of the linear economy—which is not limited to plastic, but plastic was used as a demonstrative example. The debate refocused public attention on the environmental consequences of industrialization and generated what we consider a positive momentum for change. Plastic, and more specifically microplastic, has been considered a good vehicle to communicate environmental sustainability issues to the public^59^. However, while there are a number of ways to use governance to reduce or shift plastic consumption, use, and disposal practices, for the case of microplastics, it is not clear to us if technical bans are the route to significant reductions of microplastics in the environment. In our view, a major part of the issue can and must be prevented by proper (macroplastic) waste collection, ideally as part of the transition towards a circular economy. This would help to solve the issue of secondary microplastics, which originate from the fragmentation of plastic waste dispersed in soil and water. In comparison, the issue of primary microplastics, which are already produced in the form of small particles, is a relatively minor emission source yet this is the only material which falls within the scope of proposed regulations for microplastics specifically.

With the new REACH regulations, microplastics are now an issue of chemical risk management. Previously, REACH regulation established the principle of “no data–no market” to ensure safe use of chemicals. Original REACH regulations categorically excluded polymers from the registration regime as they were considered not bioavailable due to their high molar mass. However, the conversation has now become more nuanced, with regulations that can individually cover polymer, additives, and inclusion of (primary) microplastics (Table 1). At the molecular level, the additives used in polymers are small molecules subject to REACH registration, and thus need to be tested for their human and environmental hazard and exposure profiles. The US-EPA (Environmental Protection Agency (USA)) and most other non-EU jurisdictions apply the concept of polymers of low concern (PLCs), which are “those deemed to have insignificant environmental and human health impacts”^24^. Polymers, including solid plastics, are considered PLC if they meet specific criteria related to molar mass and low-molar-weight compounds (below 1000 and 10,000 g/mol) and if they do not meet exclusion criteria, such as pre-defined reactive functional groups or chemical elements (incl. fluorines), (bio)degradation and/or water solubility, and cationicity (Supplementary Table 1). The solid plastics produced in high tonnage are PLCs by all criteria, with the exception of PVC.

### Is the microplastic definition fit for purpose?

**Fig. 3:**
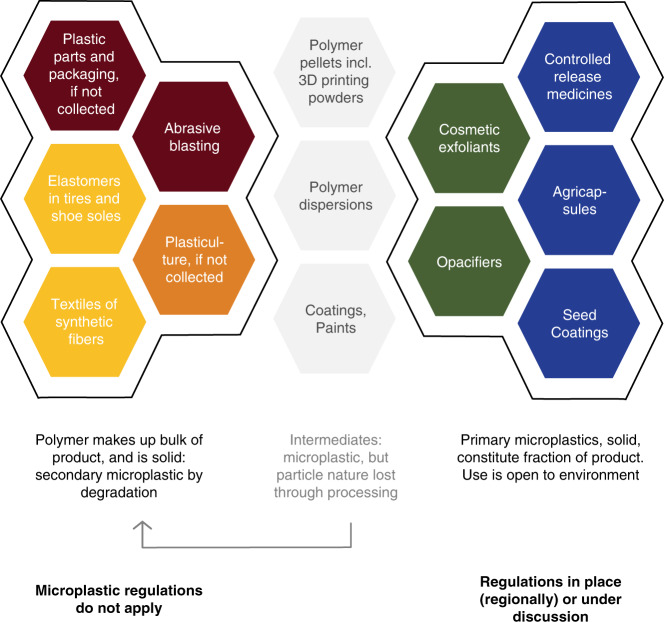
Boundaries of microplastic regulation. Bans on primary microplastics are in place or imminent across many jurisdictions, aiming to stop pollution from targeted environmental applications, from particles that are lost through consumption or targeted environmental applications (green and blue hexagons). Some industrial intermediates (gray hexagons) may contain microplastics, but these particle forms vanish through further processing or product use. Labeling and reporting requirements on intermediates may be motivation for the development of label-free alternatives, if the regulatory definitions are precise and enforceable. Microplastic regulations do not aim to resolve the dominant sources of microplastic pollution, which instead require interventions through waste management or product reformulation (red, yellow and orange hexagons). Note hexagon coloring is linked to environmental microplastic sources in Fig. 2.

The ECHA proposal for a restriction of primary microplastics uses a complex definition. The scope includes products containing more than 0.01% of microplastic particles, which are defined as particles containing solid polymer, whereof more than 1% are below 5 mm in size. A lower limit of 100 nm is being debated^60^. The scope also includes fibers of length between 3 nm to 15 mm, particles of any composition with a polymer content of ≥1% or with a continuous polymer surface coating of any thickness. The scope excludes polymers that are biodegradable or natural or soluble. All products within the scope of this complex definition will need to be labeled, and their uses to be reported to ECHA. This restriction includes conventional materials that are intermediate industrial products, such as plastic pellets and polymer dispersions (Fig. 3). In our opinion, this will generate a considerable administrative burden without realistically reducing pollution. Products containing particles that do not lose their particle nature through processing will be banned, except for some very specific derogations based on other means of technical containment. The ban thus mainly targets cosmetic beads, agricultural capsules and seed coatings (Fig. 3). This approach aims to reduce about 42,400 tons of yearly plastic release to the environment^60^, though it remains to be seen if the reduction from these sources alone will be considered substantial as long as the contributions from tire wear, textile shedding, and waste fragmentation continues to prevail in far higher tonnages.

The analytical challenges of adhering to the complex definitions prescribed by ECHA are enormous, and for any polymer-containing product, one would have to assess solidity, solubility and the continuity of coatings for traces down to 0.01% of particles containing polymer. Given the current analytical capabilities to measure and characterize microplastic^61^, we question how one could possibly assess the variety of products under standardized conditions at regular time intervals. In consequence, this definition is not easily enforceable. This also means that companies do not know which properties to avoid, unless opting only for polymer-free formulations (as outlined in the section “The diversity of polymers through their life cycle complicates microplastic risk assessment”). Avoiding polymers all together would also limit use of functional polymers (Fig. 1), which is contrary to the intentions of ECHA. The original draft of the restriction gives rather undirected incentives, because the lack of enforceable criteria effectively widens the scope to all polymers. The restriction would become easier to enforce, and more precisely targeted to the issue of microplastics, if a measurable lower size limit of 100 nm was introduced and if the scope was focused to solid and insoluble polymers. Both these measures would help to clarify that functional polymers (Fig. 1) are not in scope of the microplastic definition. The lower size limit, the solubility, and the biodegradability criteria are currently a matter of debate and political negotiation^60^. ECHA’s proposal set out specific test methods and pass criteria for identifying biodegradable polymers, which are excluded from the restriction. A high-profile committee, the Risk Assessment Committee (RAC), concluded on criteria for defining biodegradability of particulate polymers even though some standards, e.g. OECD testing guidelines, are still not suited to cover the properties of particulates. Precise and measurable criteria for biodegradability provide the opportunity to incentivize biodegradable solutions where they make sense, based on an assessment that considers the intended uses, for which biodegradation can be designed, tested and enforced.

Scientists still need to test hypotheses about which aspects of microplastics (polymer, molecular structure, additives, physical–chemical-biological interactions, size: summarized in Table SI_1) are most responsible for causing adverse effects. Nevertheless, we have the impression that the debate on microplastics has provided a welcome bridge towards the registration of polymers under REACH, following suit to the K-REACH example (Table 1). Articles made of polymers for specific purposes already have to fulfill sector-specific regulation, e.g., for food contact materials, toys, medical devices, etc., where migration of additives and absence of oligomers are key criteria^62,63^. In any case, producers are responsible for the safe use of their products, and polymers-requiring-registration require notification to the Classification and Labeling inventory. In the future, they will have to demonstrate safe use via REACH registration, at least for some polymers. In this context, we need to carefully weigh the costs of taking a precautionary approach as well. It is also important to note that not taking a precautionary approach to the use of microplastics for all purposes is not advocating to wait for other plastic reduction measures^64^. We whole-heartedly support initiatives and innovation which reduces unnecessary plastic waste, improves waste disposal streams, smart uses of biodegradable polymers, and increases in material circularity (including recycling efforts). Issues of primary microplastic and macroplastic, while interlinked at some level, may still be considered separately.

## Towards the identification of priority regulatory cases—innovative new materials and protection of environmental health

While bans and prohibitions of plastics are so far the central reactions of governments to anti-plastic norms, there does not have to be an all or nothing approach when it comes to microplastic use. Alternatively, regulations may be considered a catalyst for change and a nudge towards further development of new and improved practices^65^. Initiatives to ban release of microplastics that rely on corporate societal responsibility may leave as many loopholes as a non-enforceable regulation^66^. We believe that only an easily measurable definition of microplastic can minimize false claims and spread of misinformation to consumers. Recognized certifications will ultimately support and reinforce industry social responsibility and social license.

It is important to keep in mind that many plastics are actually quite effective problem solvers during their life cycle (e.g. food safety from packaging), and such applications are not always easily replaced and/or alternative materials may not have better sustainability scores. However, further innovation is possible. Within the framework of Green Chemistry (the practice of designing chemical products and processes which reduce or eliminate the use or generation of hazardous substances), the use of polymers may, in some cases, promote and enhance solutions to economic and sustainability goals. As one example of how Green Chemistry may help to mediate one plastic pollution source, biodegradable mulch films are reducing (micro)plastic waste in agricultural fields, which we believe is a positive innovation in that sector. Biodegradability is a certified performance characteristic and is not dependent on the origin of raw materials, but only on the chemical composition of the polymer. Bio-based polymers are not necessarily biodegradable, and vice versa. Biodegradable materials have been available from the chemical industry for some time, yet today they are still a more expensive alternative to polyethylene (PE), and so they have not been as attractive as an option in many circumstances. Very clearly, biodegradability is no excuse for littering. Biodegradation needs to be specified and tested for specific intended uses, and biodegradable plastics are not an overarching solution for all pollution by plastic waste. But, they can be a solution to issues of plastic and primary microplastic that is intentionally used with known pathways into the environment. With the current debate on plastic use, if consumers are willing to pay a premium for biodegradable plastics and regulations target key industries to limit conventional polymers, biodegradable products may become more competitive with conventional alternatives. In this way, we foresee that regulation can spur industries and consumers to develop and establish more sustainable alternatives, even if they are more expensive in the short term.

As a main alternative to abstaining from plastic usage, businesses often focus on recycling as a viable choice be part of the solution to reduce plastic burdens in the environment. Initiatives like “This is Plastics” or “Marine Litter Solutions” lobby for better waste management infrastructure (especially in the Global South). Consumers are also encouraged to recycle, e.g. through the “I Want To Be Recycled” campaign from the joint partnership between the environmental NGO, “Keep America Beautiful” and corporations from the plastics, packaging, and food industries. On one hand, this allows citizens the opportunity to feel involved with finding solutions to the plastic waste issue, even though this puts (a portion of) the burden of sustainability back with the consumer, instead of with corporations or governments. However, not all plastics products fit into plastic recycling schemes, in addition to the issues of additives and blends that are used in products^67^, or issues of soiling and improper sorting. Reliance on recycling facilities in certain regions can also lead to disturbances in global plastic waste management, as seen when China stopped the import of nonindustrial plastic waste in 2017 (ref. ^68^). Nevertheless, plastics recycling is experiencing a renaissance. Some recycling routes (e.g. pyrolysis feedstocks) destroy the polymer altogether, whereas recycling by material feedstock retains the polymer molecules, and shreds plastic parts to particles before sorting. Because of these various treatment chains, we think that the regulatory incentives in a circular economy must acknowledge that chemcycling via pyrolysis and thermal recycling can be just as sustainable as the seemingly simpler materials recycling, which suffers from technical downgrading due to impurities and mixed additives. From the example of recycling, we can learn that there may not be one hard and fast rule as to what is best in terms of plastic governance.

A cost–benefit approach to evaluate the societal and environmental impacts of primary microplastic restriction is warranted, and is currently ongoing as regulations are developed. What is less clear is how to position individual microplastic applications along the cost–benefit value chain. The balance of these pros and cons may be very subjective, and the necessity and suprifilousness of a given microplastic use will be different for different stakeholders. In some situations, such as microbeads in rinse-off products, societal benefits are negligible, inexpensive alternatives exist, and the public, government, and industry by and large support discontinued use. However, decisions will become more complicated for products or processes which involve microplastics which are not so readily replaced or have more defendable benefits. Encapsulated fertilizers and plant protection products may now be banned under new REACH restrictions for primary microplastics, even though they have proven benefits of having a more targeted release of active ingredients and thus ultimately leads to less fertilizer or pesticide in the soil. There is anticipated to be a 5–8-year allowance to reformulate materials to comply with new regulations, although it realistically takes between 10 and 15 years to bring new formulations to market. In a different industry segment, the replacement of metals by plastics is a success story for automotive engineering, and is indispensable in curbing the CO_2_ footprint of the automotive fleet. Additive manufacturing is the next step of optimized structure and delocalized production, but now all solid plastic pellets below 5 mm are to be labeled and reported, with increased regulatory pressure on the micronized powders. These are just two examples of the cost–benefit analysis of microplastic usage, where we do not see that more stringent regulations on microplastics necessarily serves the stated goal of improving environmental health.

Plastic pollution is quite clearly a critical environmental problem on a global scale, and can be tackled on a number of fronts, including reduction of plastic use, inclusion of plastic into the circular economy, and better waste management^69,70^. Out of the list of six sources of solid microplastic pollution (Fig. 2), the critical cases that need incentives for innovation by regulation are those with intended open uses. This is not to say that other cases are less important, but they may be more effectively dealt with by different approaches than through the type of regulations that ECHA are currently proposing. Plastic from intended open use (e.g. uncollected mulch films) and primary microplastic from intended open use (e.g. seed coatings) are two such examples. Here, we believe industry has to improve the sustainability of current solutions, and biodegradability (which is the key exemption in the proposed ECHA restriction) is likely the key to success—if ECHA’s biodegradation criteria remain achievable and adapted to the intended use. Yet we also urge caution when putting pressure to adopt greener alternatives. Biodegradable plastics have been touted as a key alternative to reduce plastic usage, but not all intended uses of polymers allow for biodegradability for the products to function along their life cycle, such as polymer binders used in coatings, paints, and adhesives, or polymer pellets that are molten and injection molded to automotive parts, as examples. This also suggests that regulations targeting other sources of primary microplastic pollution (e.g. transportation losses) will need to rely on strategies which do not involve biodegradability as the core component.

## Future outlook

The accumulation of plastic in the environment will ultimately have an impact on water and soil quality, and so a sustainable relationship with plastic is a necessity for the Anthropocene. While plastics, and microplastics, have been a uniquely successful lighting rod to activate the public on matters of pollution and sustainability, we cannot let that drive our desire to continue making environmental gains through plastic restrictions alone. Many years of research have gone into the materials we currently use, and thus their physical/chemical properties, and costs, are optimized from the point of view of manufactures. Plastic opponents criticize plastic production and use because of all the externalities and impacts that we cannot fully characterize and control. With additional research and development, alternative materials may catch up in terms of both price and performance, but our limited global resources should be targeted to scientifically defendable cases of increased sustainability^71^, not to regrettable replacements or marketing stories. Therefore, we need an unbiased assessment of the hazard, fate, and societal benefits of primary microplastics throughout the regulation process. Regulation should be enforceable and focused, and most importantly linked to hazards. Then the replacement of critical microplastics can become an example of sustainable development and strict environmental regulations can stimulate innovation of new, more competitive and environmentally conscious materials.

## Supplementary information

Supplementary Information
