# Supplementary material for: Microplastic regulation should be more precise to incentivize both innovation and environmental safety
Source: Nat Commun. 2020 Oct 21;11:5324. doi: 10.1038/s41467-020-19069-1 (PMC7578016; doi:10.1038/s41467-020-19069-1)
Supplement: Supplementary file 1 — Supplementary Information [file 41467_2020_19069_MOESM1_ESM.pdf]

Supplementary Information for Microplastic regulation should be more precise to incentivize both innovation and environmental safety by Mitrano and Wohlleben

Supplementary Note

Considering that microplastics consist of polymers, the descriptors of molecular structure and physical-chemical interactions as used by the OECD concept of polymers of low concern (Table SI_1), and of the ECETOC risk assessment framework for polymers are relevant.[1] Considering the analogy to nanoparticles, the descriptors for REACH registration of nanoforms[2-5] may serve as a baseline to form an initial hypothesis of properties that are likely relevant to assess the toxicity of solid particulate plastics (Table SI_1, shaded blue).[6-8] The fact that plastic can reach remote sites by aerosol transport,[9] suggests that inhalation, which is the most critical human exposure route for nanoparticles, is relevant for microplastics as well.[10] The description of chemical composition would require careful consideration of additives. Additives are subject to REACH registration in and of themselves (main text, Table 1). The leaching of additives from polymers is not a new issue, but it may turn out to be the decisive contribution to the hazard posed by solid plastic particles.[6,11] Compared to other small solid particles (nanomaterials, minerals, combustion particles), the leaching of chemically well-defined additives is characteristic to microplastics. However the issue of migration and hazard of additives is generic to all plastics parts and plastic particles of all sizes, and will become more important as recycling quotas increase.[12] Regulations and analytical methods are well developed to assess and approve additives in general, and especially in sensitive scenarios such as food contact packaging.[13] Plastic materials intended for food contact in the EU are regulated in regulation EU No 10/2011.[14] There are notably some contradictions in the regulations between polymers and microplastics, for example, that the absence of (bio)degradation is an inclusion criterion for PLC, whereas it triggers exemption from the proposed REACH restriction of primary microplastics (Table SI_1).

Currently, we find that the properties of the ECHA restriction bear more resemblance with the criteria for nanoform registration than with criteria of polymer assessment. Scientists still needs to test the hypotheses on risk assessment of microplastic that are summarized in Table SI_1, aiming to rank properties by their relevance for environmental and human risk assessment. And ultimately to discard as many properties as possible to further simplify this matrix for risk screening and safer-by-design product development. Precautionary action can then be replaced by targeted regulation of those microplastics that pose significant concern.

Supplementary Table 1: Physical-chemical approaches to risk assessment of microplastics. Cells shaded blue for properties that are used to assess particulate plastics from the perspective of their polymer nature (OECD, ECETOC),[1] and from the perspective of their particle nature (ECHA, REACH).[3] In this consolidated presentation, the properties can have slightly different descriptors in the different perspectives, as marked by comments where necessary.

	**Property**	**OECD Polymers of low concern (incl. non-solid)**	**ECETOC CF4polymer (non-solid polymers)**	**ECHA Microplastic restriction (draft, solid polymers)**	**REACH Nanoform registration**
**Molecular descriptors**	Chemical composition incl. NIAS & IAS	(fluorines)	(composition)	(composition)	(composition)
	Molecular weight				
	Molecular mobility or crosslinking	(linked to swelling)			
**Particle morphology descriptors**	Crystallinity		glass transition temperature		
	Particle size distribution			5 mm cut-off	
	Shape (morphology)				
	Diameter and length distribution				for fibres
	Rigidity (scales with diameter[4])				for fibres
	Solidity				(implicitly assumed)
	Density				
**Particle interaction descriptors**	Reactive functional groups				
	Surface (re)activity	(Low priority)			(radicals)
	Surface area				
	Surface-exposed functional groups				(via surface treatment)
	Surface hydrophobicity				(instead of log k_{ow})
	Surface charge / Cationicity		(instead of log k_{ow})		(instead of log k_{ow})
	Surface tension				
	Dustiness, respirable fractions				
	Transformation by biodegradation	No degradation allowed		persistence-based restriction	
	Transformation by biodissolution				
	Log k_{ow}				
	Water solubility, extractability				

Supplementary References

1 ECETOC. The ECETOC Conceptual Framework for Polymer Risk Assessment (CF4Polymers). *ECETOC Technical Report* **133-1** (2019).

2 Wohlleben, W. *et al.* The nanoGRAVUR framework to group (nano) materials for their occupational, consumer, environmental risks based on a harmonized set of material properties, applied to 34 case studies. *Nanoscale* **11**, 17637-17654 (2019).

3 Commission, E. in *C/2018/7942* (ed European Commission) (2018).

4 ECHA. How to prepare registration dossiers that cover nanoforms: best practices. doi:10.2823/128306 (2017).

5 ECHA. Appendix R.6-1 for nanomaterials applicable to the Guidance on QSARs and Grouping of Chemicals. doi:10.2823/884050 (2017).

6 Hartmann, N. B. *et al.* Are We Speaking the Same Language? Recommendations for a Definition and Categorization Framework for Plastic Debris. *Environmental Science & Technology* **53**, 1039-1047, doi:10.1021/acs.est.8b05297 (2019).

7 Hüffer, T., Praetorius, A., Wagner, S., Von Der Kammer, F. & Hofmann, T. Microplastic exposure assessment in aquatic environments: learning from similarities and differences to engineered nanoparticles. *Environmental Science & Technology*, doi:10.1021/acs.est.6b04054 (2017).

8 Andrady, A. L. The plastic in microplastics: a review. *Marine pollution bulletin* **119**, 12-22 (2017).

9 Bergmann, M. *et al.* White and wonderful? Microplastics prevail in snow from the Alps to the Arctic. *Science advances* **5**, eaax1157 (2019).

10 Vianello, A., Jensen, R. L., Liu, L. & Vollertsen, J. Simulating human exposure to indoor airborne microplastics using a Breathing Thermal Manikin. *Scientific reports* **9**, 1-11 (2019).

11 Zimmermann, L., Dierkes, G., Ternes, T. A., Völker, C. & Wagner, M. Benchmarking the in Vitro Toxicity and Chemical Composition of Plastic Consumer Products. *Environmental Science & Technology*, doi:10.1021/acs.est.9b02293 (2019).

12 Geueke, B., Groh, K. & Muncke, J. Food packaging in the circular economy: Overview of chemical safety aspects for commonly used materials. *Journal of Cleaner Production* **193**, 491-505, doi:https://doi.org/10.1016/j.jclepro.2018.05.005 (2018).

13 Hoppe, M., de Voogt, P. & Franz, R. Identification and quantification of oligomers as potential migrants in plastics food contact materials with a focus in polycondensates – A review. *Trends in Food Science & Technology* **50**, 118-130, doi:https://doi.org/10.1016/j.tifs.2016.01.018 (2016).

14 European_Commission. COMMISSION REGULATION (EU) No 10/2011 of 14 January 2011 on plastic materials and articles intended to come into contact with food. *Official Journal of the European Communities.* (2011).
